# Elucidation of chemical profiles and molecular targets of *Mondia whitei* leave fractions bioactive as novel therapeutics: an in vitro and in silico assay

**DOI:** 10.1186/s43141-022-00440-2

**Published:** 2022-12-27

**Authors:** Hope Onohuean, Fanny Eseohe Onohuean, Sharon Iyobor Igbinoba, Joseph Obiezu Chukwujekwu Ezeonwumelu, Peter Chinedu Agu, Josiah Eseoghene Ifie, Tusubira Deusdedit, Patrick Maduabuchi Aja

**Affiliations:** 1 Biomolecules, Metagenomics, Endocrine, and Tropical Disease Research Group (BMETDREG), Kampala International University Western Campus, Ishaka-Bushenyi, Uganda; 2 Biopharmaceutics Unit, Department of Pharmacology and Toxicology, Kampala International University Western Campus, Ishaka-Bushenyi, Uganda; 3 Department of Clinical Pharmacy and Pharmacy Administration, Faculty of Pharmacy, Obafemi Awolowo University, Ile-Ife, Nigeria; 4 Department of Clinical Pharmacy, Kampala International University Western Campus, Ishaka-Bushenyi, Uganda; 5 Department of Biochemistry, Faculty of Biological Sciences, Ebonyi State University, Abakaliki, Nigeria; 6 Department of Medical Biochemistry, Faculty of Biomedical Sciences, Kampala International University, Kampala, Uganda; 7 Department of Biochemistry, Faculty of Medicine, Mbarara University of Sciences and Technology, Mbarara, Uganda

**Keywords:** *Mondia* w*hitei*, Molecular targets, Novel therapeutics, In vitro, In silico assay

## Abstract

**Background:**

*Mondia whitei* root is often used in Africa as a local therapeutic agent for libido enhancement. The fractions of the *M. whitei* leaves (MWL) lack chemical characterization of their bioactive components and possible molecular targets. We characterized and investigated its molecular target as therapeutic agents in an in vitro and in silico assay. Mineral compositions, antioxidant, and GC-MS characterization were studied. The cytotoxicity effect was measured on HeLa and HT-29 cells by MTT assay. In silico potential inhibitors of Cathepsin B (CathB) as a cancer biomarker were determined.

**Results:**

The flame photometry produced marked Na^+^ and K^+^. GC-MS revealed eighteen bioactive components. The fractions (chloroformic 47.00, ethanolic 45.52, and aqueous 40.13) of MWL caused a higher inhibition ratio compared to standards. The MWL showed a significant cytotoxic effect on the treated cell lines at concentrations of 150 and 200 μg/ml and 100, 150, and 200 μg/ml for HT-29 and HeLa cells, respectively. Ten bioactives (MWL 4, 5, 6, 8, 9, 10, 14, 15, 17, and 18) showed potential inhibition of CathB with binding affinities of −4.40 to −8.3 Kcal/Mol. However, MWL 4, 9, 14, and 17 which have higher binding affinities (−6.7, −7.1, −8.2, and −8.3, respectively) than the standard inhibitor (−6.5) were the lead molecules.

**Conclusion:**

These chemical profiles and potential molecular targets unraveled in this study propose that MWL has a promising anticancer activity.

**Graphical Abstract:**

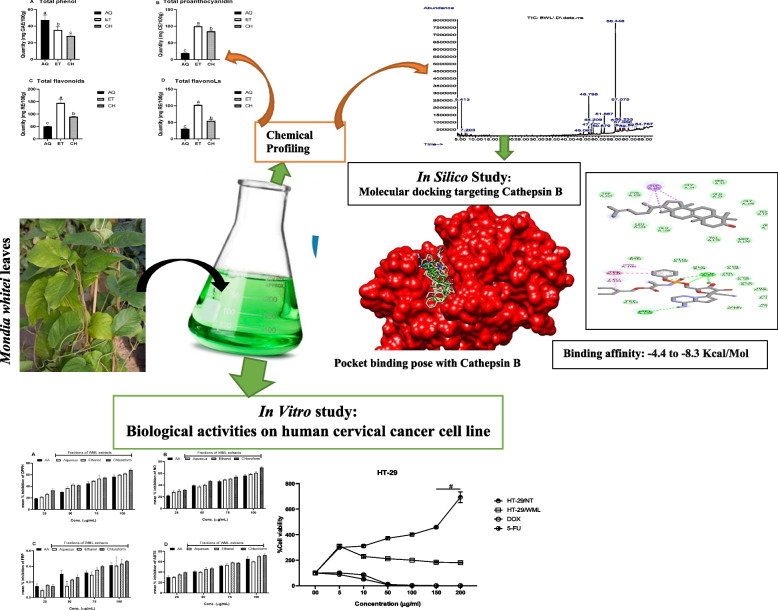

## Background

Reactive oxygen species (ROS) and free radicals are essential biochemical byproducts required for cell signaling and vital physiological processes. But excessive production and unhinging amounts are the basis for alteration in cellular redox equilibrium, which ultimately leads to disruption of normal biological functions. Subsequently, an imbalanced cellular mechanism among activities of ROS and antioxidant/scavenging defense systems results in oxidative stress (OS) [1]. OS and free radical arbitrated reactions are implicated in the pathogenesis, progression, and severity of many diseases such as cancer [2–5], cardiovascular diseases including atherosclerosis [6], diabetes [7, 8], neurodegenerative diseases [9], and aging [10], which are significant causes of death globally [11] and whose underlying mechanisms are poorly understood until date.

Over the past decades, evidence has shown that the overproduction of these reactive molecules facilitates oxidative damage to cellular structures and components such as proteins, lipids, and DNA [12, 13]. For instance, ROS and reactive nitrogen species (RNS) are linked to inflammation of the liver resulting in chronic hepatic cell damage and fibrosis [14], and excessive nitric oxide (NO) radicals generated via the NO synthase pathway are implicated in the induction of nitrosative stress [15]. Additionally, NO**·** inhibits protein synthesis and interrupts lipid metabolism. It binds to superoxides and generates toxic peroxynitrite that causes or induces hepatic cell degeneration [16].

Cathepsin B (CathB), a lysosomal cysteine protease, is involved in numerous human disease mechanisms through its translocation from acidic lysosomes to neutral pH of cellular locations, including the cytosol and extracellular environment [17]. CathB mediates the regulation of oxidative stress [18]. Inhibition of CathB activity maintains the function of mitochondria and decreases the generation of ROS during in vitro aging of oocytes [19]. Genetic ablation of CathB in mice significantly decreases the generation of ROS during neuroinflammation and improves cognitive impairment during aging. The CA-074 methyl ester is a selective inhibitor of CathB [20] and has been utilized in numerous studies to demonstrate the role of CathB protease in cellular and physiological functions [21]. Thus, CA-074 was used in the present study as a standard inhibitor to predict the precise binding site of CathB.

One of the significant defensive mechanisms against radical-induced oxidative and nitrosative stress is the nonenzymatic antioxidant defense mechanism. Plant foods, vegetables, and fruit are highly rich in antioxidant properties, which could help prevent chronic diseases caused by oxidative stress. Plant phytochemicals such as flavonoids, carotenoids, phenolic acids, and terpenoids are indicated to be responsible for their enormous antioxidant properties [21–23].

*Mondia whitei* leaves (MWL) (Hook. f.) belong to the Apocynaceae family and is an endemic African plant species except in the northern part of the continent. *M. whitei* is locally called “Ado” in Central Igboland (Aguata) of Anambra state and “Akoro” in northern Igboland (Abakaliki) of Ebonyi state, which are in the southeastern part of Nigeria. It has been helpful to the populace since ancient times [24]. Generally in Africa, the roots of *M. whitei* have been used traditionally as an aphrodisiac and also in the management of sexual weakness (asthenia), erectile dysfunction, premature ejaculation, and an increase in sperm production [24–30]. Other traditional uses include appetite stimulation and management of stomach pain, body pain, indigestion, gastrointestinal disorders, gonorrhea, postpartum bleeding, pediatric asthma, and vomiting [31]. In addition, different parts of the plant are used to treat diverse ailments such as indigestion, a tonic, appetite stimulant, antacid, urinary tract infection, jaundice, headache, and diarrhea [32]. The leaves, fruits, and roots of *M. whitei* are generally used in West Africa (southeastern Nigeria) as food spices and for flu and cough treatment; in Cameroon as a sports drink and aphrodisiac; in Zimbabwe for the treatment of anorexia, bilharzia, appetite, and libido, as a galactagogue, fertility medication, and as an antidepressant [33]; in Rwanda for treatment of diarrhea [31]; and as a stimulant and for erectile dysfunction in southwestern Uganda [28].

Despite the usefulness of this plant in treating myriad diseases, plausible mechanisms that may be responsible for the observed effects are yet to be elucidated, both in vitro and in vivo. Also, there is no comprehensive quantitative phytochemical analysis, macro and micronutrient screening of its leaf fractions, and there is a dearth of information on the bioactive compounds in the leaf fractions. This leaves us with the challenge of conducting more stringent research to isolate and identify the species’ novel bioactive compound (s). Because of this, we demonstrated chemical profile, antioxidant and cytotoxic effect, as well as further virtually screened the bioactive constituents for potential inhibitors of CathB which has been linked to various kinds of cancer progression through mediating oxidative stress.

## Methods

### Experimental plant collection and authentication

MWL were harvested from KIU-Western Campus staff quarters garden Ishaka-Bushenyi and authenticated by a taxonomist, and the identification specimen number (no. HOPE-PHA-2018/001) was deposited in the School of Pharmacy Herbarium, Kampala International University (KIU), for future citation/references.

The fresh leaves were washed on a running tap and air-dried at room temperature for weeks, crushed into powder using a clean mortar and pestle, and stored in the desiccator until ready for use in the Department of Pharmacology Laboratory, KIU.

### Extraction and fractionation of MWL

MWL ethanolic fractions were extracted as described [34, 35]. Briefly, 300 g of the plant powder was macerated in 1.5 l of absolute ethanol and agitated every 12 h for 4 days. The solvent was filtered and concentrated in a rotary evaporator and oven-dried at 40 °C overnight. From the crude ethanolic extract, 70 g was dissolved in 150 ml of distilled water, shaken vigorously to ensure proper mixing, and then transferred to a separating funnel. About 1 l of chloroform was added, shaken, and left overnight. The sample was partitioned, having the chloroform partitioned down the separating funnel, while the aqueous fraction remained at the top. The chloroform fraction was carefully collected, concentrated, and oven-dried at 40 °C and stored until needed. The aqueous fraction left was dried in the oven at 40 °C over 1 day.

### Chemical profiling

#### Determination of macro/micronutrient content of MWL extracts

The MWL extract fractions (aqueous, ethanol, and chloroform) were analyzed for the presence of trace elements referred to as macro/micronutrients of medical importance. An atomic absorption spectrophotometer (AAS 969 Unicam Solar 32) was used to identify and quantify the essential mineral content of extract fractions by adopting the protocols of AOAC, 2000, while flame photometry (JENWAY PF7) was specifically used to evaluate Na+ and K.

#### Determination of phytochemical content of MWL extracts

The analysis of phytochemicals such as phenolic, flavonoid, saponins, tannins, steroids, alkaloids, anthraquinones, terpenoids, triterpenoids, reducing sugar, and cardiac glycoside was determined by standard methods reported by Sofowora [36], Trease and Evans [37], and Harborne [38, 39].

#### Determination of GC-MS profile of MWL extracts

An Agilent 7890B GC System coupled with 5977A MSD was used to analyze the active constituents of MWL fractional extracts. The data analysis software was MassHunter; column specification: HP-5 fused silica capillary column (30 m × 0.320 mm i.d. and 0.250 μm film thickness); inlet: 250 °C (temp.) and 48.745 kPa (pressure) operated at pulse splitless mode; injection volume: 1 μl, carrier gas: helium (99.999% purity); average velocity: 36.262 cm/s; auxiliary temp: 280 °C; oven temperature programming: start from 40 °C (held for 1 min); ramped to 240 °C at 3 °C/min; and total runtime: 67.667 min [40].

### In vitro study

#### Determinations of antioxidant activities of MWL extracts

##### Nitric oxide scavenging activity assay

A 2 ml of 10 mM sodium nitroprusside dissolved in 0.5 ml phosphate buffer saline (pH 7.4) was mixed with 0.5 ml of MWL extracts at various concentrations (0.25, 0.50, 0.75, and 1.0 mg/ml). The mixture was incubated at room temperature for 150 min. Then, 1 ml of incubated solution was mixed with 1 ml of Nedd reagent. The mixture was incubated at room temperature for 30 min. Absorbance was measured using a spectrophotometer at 540 nm. Ascorbic acid was used as standard. Blank was 1 ml of water, 2 ml of sodium nitroprusside, and 1 ml of Nedd reagent. Control was 2 ml of sodium nitroprusside, 0.5 ml of phosphate buffer, 1 ml of Nedd reagent, and 0.5 ml of methanol. The number of nitric oxide radicals scavenged was determined using the formula shown below:

$$\%\textrm{Inhibition}=\left[\left(\textrm{Ac}-\textrm{As}\right)/\textrm{Ac}\right]\ast 100$$

where Ac = absorbance of the control and As = absorbance of the plant extract.

##### 2,2-Diphenyl-1-picrylhydrazyl (DPPH) free radical scavenging activity assay

The DPPH radical scavenging activity was determined according to the procedure previously described [41]. At various concentrations, an aliquot of 2 ml of 0.04% DPPH solution in ethanol and 1.0 ml of MWL extracts/garlic acid were mixed. The mixture was shaken vigorously and allowed to reach a steady state at room temperature for 30 min in a dark chamber. Decolorization of DPPH was determined by measuring the absorbance at 517 nm. A control was prepared using 1 ml ethanol mixed with 2 ml of DPPH. The percentage inhibition of DPPH radicals by the extract/compound was determined by comparing the absorbance values of the control and the experimental tubes. The percentage of inhibition was calculated using the following:

$$\%\textrm{Inhibition}=\left[\left(\textrm{Ac}-\textrm{As}\right)/\textrm{Ac}\right]\ast 100$$

where Ac is the absorbance of control and As is the absorbance of extracts/ascorbic acid.

##### Ferric reducing power activity

The Fe^3+^ reducing powers of the three MWL extracts were determined as previously reported with slight modification [34, 42]. Different concentrations (25–100 μg/ml) of the extracts (1.0 ml) were mixed with 2.5 ml phosphate buffer (pH 7.0) and 2.5 ml potassium ferricyanide (1%), followed by incubation at 50 °C for 30 min. After incubation, 2.5 ml of trichloroacetic acid (TCA, 10%) was added to terminate the reaction and centrifuged at 3000 rpm for 10 min. The upper portion of the solution (2.5 ml) was mixed with 2.5 ml of distilled water, and 0.5 ml of FeCl_3_ solution (0.1%) was added. The reaction mixture was left for 10 min at room temperature, and the absorbance was measured at 700 nm against an appropriate blank solution. Ascorbic acid was used as a positive control/standard, and all tests were performed in duplicate. Higher absorbance of the reaction mixture indicated greater reducing power [43].

##### ABTS radical scavenging activity

To determine the antioxidant capacity of ABTS radical (ABTS^+^), we adopted the method described by Oyedemi et al. [34]. ABTS radical was prepared from 7 mM ABTS stable radical aqueous liquid with 2.45 mM K_2_S_2_O_8_ in ratio 1:1 and kept away from light for 12–18 h at 27 °C. The absorbance was adjusted to 0.800 ± 0.004 at 734 nm by diluting the ABTS solution with methanol (1:50 v/v) before the commencement of the assay. MWL fractions and standard control of varying concentrations (25–100 μg/mL) were dissolved in 1 ml of ABTS in test tubes in a dark chamber for 7 min, and the absorbance was measured at 734 nm against the ethanol (blank). The percentage inhibition of samples and standards was expressed as follows:

$$\%\textrm{ABTS}\ \textrm{scavenging}=\left[\left(\textrm{Ac}-\textrm{As}\right)/\textrm{Ac}\right]\ast 100$$

where Ac is the absorbance of control and As is the absorbance of extracts/ascorbic acid. IC_50_ value (a concentration at 50% inhibition) was determined from the curve between percentage inhibition and concentration. All determinations were done in duplicate, and the IC_50_ value was calculated by using the equation of the line.

#### Determination of cytotoxicity effects of MWL extracts

HeLa cervical adenocarcinoma cells (Highveld Biological (Pty) Ltd. Johannesburg, SA) and HT-29 (human colorectal adenocarcinoma) Cellonex (South Africa, were cultured in RPMI-1640 media or Dulbecco’s Modified Eagle’s Medium (Invitrogen, Carlsbad, CA, USA) and supplemented with 10% fetal bovine serum, 1% penicillin/streptomycin antibiotics, and 1% l-glutamine. The cells were treated and grown in a humidified atmosphere condition of 5% CO_2_ at 37 °C; a total of 2.5 × 10^4^ cells/well were seeded in a 96-well plate for 48 h and thereafter treated at different concentrations of the ethanolic extracts of MWL and incubated for 24 h [44]. The CellTiter 96® Aqueous One Solution Cell Proliferation (MTS) (USA) was used to assay cell viability according to the manufacturer’s directions. Briefly, a total of 10 μl MTS solution per 100 μl of the treatment media was added to each well and incubated at 37 °C for 2 h. Absorbance was measured at 490 nm [45]. A linear regression curve was used to determine the IC50 values. The viability of cells as a percentage was estimated as follows:

$$\textrm{Cell}\ \textrm{viability}\ \left(\%\right)=\frac{\textrm{OD}\ \textrm{of}\ \textrm{treatment}}{\textrm{OD}\ \textrm{of}\ \textrm{control}}\times 100$$

### Statistical analysis

The antioxidant results were subjected to Tukey’s post hoc test or two-way ANOVA, and other results were subjected to the LSD post hoc test of one-way ANOVA. All statistical significance was considered at *p* < 0.001, < 0.01, and < 0.05, and graphical presentations were done in GraphPad Prism software version 8.0.2.

### In silico assay

#### Targets retrieval and preparations

**Fig. 1:**
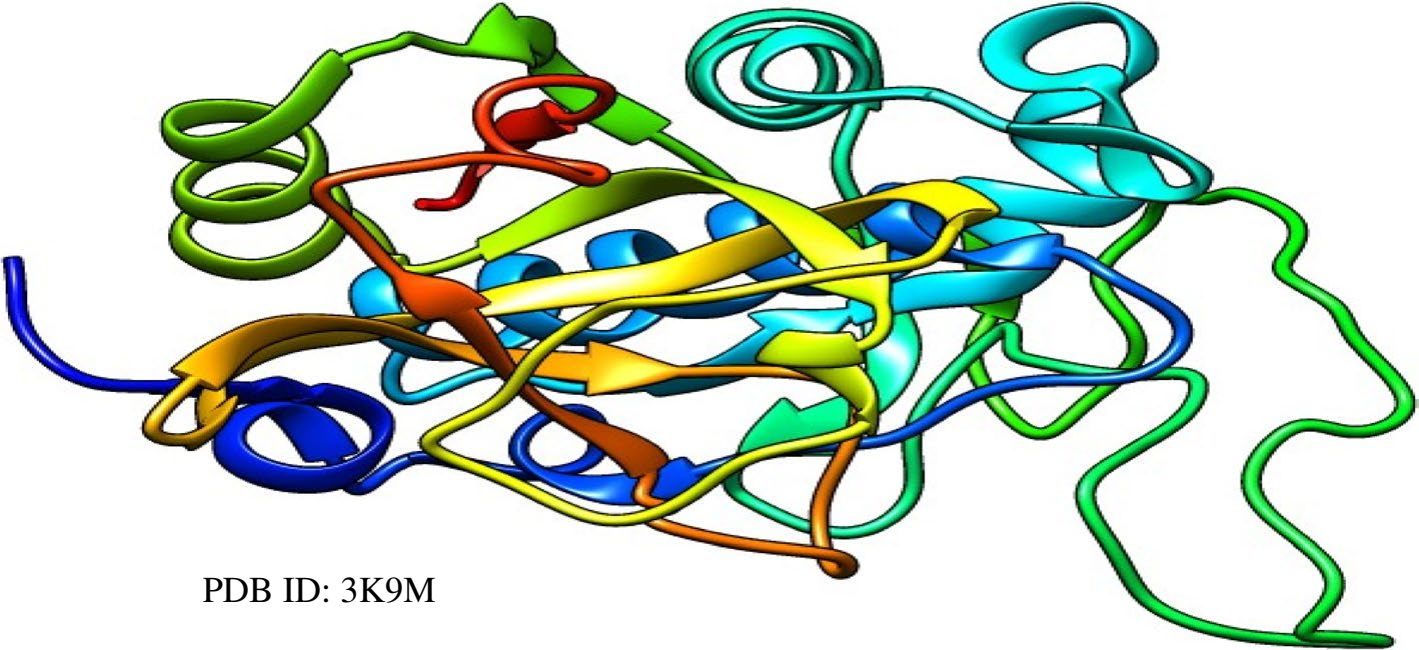
Cathepsin B. The 3D crystal structure of Cathepsin B in ribbon (visualized in UCSF Chimera)

The 3D crystal structures of the CathB (PDB ID: 3K9M) (Fig. 1), standard inhibitor, and MWL bioactive (MWL1–18) were retrieved per PubChem CID and prepared in UCSF Chimera as described in Aja et al. [46].

#### Molecular docking

Briefly, the prepared CathB was loaded into PyRx and converted to a macromolecule. The standard inhibitor (CA-074) and the MWL1–18 were imported into the PyRx and converted to pdbqt, hence minimized. The grid box was set at X, Y, and Z orientation (center: 16.0344, −2.2183, 8.9326 and dimension: 62.2728, 47.5059, 57.1066) and run at exhaustiveness of 8. We selected the docking scores at the RSMD value of zero. Only the MWL bioactive that binds in the same site with CA-074 was considered a potential inhibitor. We further identified the protein-ligand interactions of the MWL bioactives with stronger affinity than CA-074 for CathB as the lead molecules using the Discovery Studio Visualizer 2021.

## Results

### Micro/macronutrient contents of MWL extracts

The result shows a variety of micro and macronutrient present in the MWL, as depicted in Table 1. The highest amounts of sodium, potassium, iron, copper, and nickel were obtained from the ethanolic fraction. The aqueous fraction gave the highest quantities of magnesium, zinc, and manganese [47].

$$1\ \textrm{ppm}\times 10=1\ \textrm{mg}/100\ \textrm{g}\ \textrm{or}\ 1\ \textrm{ppm}=1\ \textrm{mg}/\textrm{kg}\ \left(\textrm{Convert}-\textrm{Me}.\textrm{Com}-\textrm{Online}\ \textrm{Units}\ \textrm{Conversion}\right)$$

**Table 1:** *Mondia whitei* leaves micro and macronutrients

S/no.		Aqueous	Ethanol	Chloroform
Micronutrients (ppm)	Na	24.5201	36.1021	26.2031
	K	35.5001	40.6543	19.7053
	Mg	5.0008	2.5006	3.6007
	Fe	0.5608	0.7003	0.1105
	Pb	ND	ND	ND
	Cu	0.1355	1.0003	0.9115
	Mn	0.0613	0.017	0.01405
	Zn	2.1009	0.0988	0.8403
	Cd	ND	ND	ND
	Ni	0.0122	0.0158	ND

*ND* not detected

### Phytochemical contents of MWL extracts

**Fig. 2:**
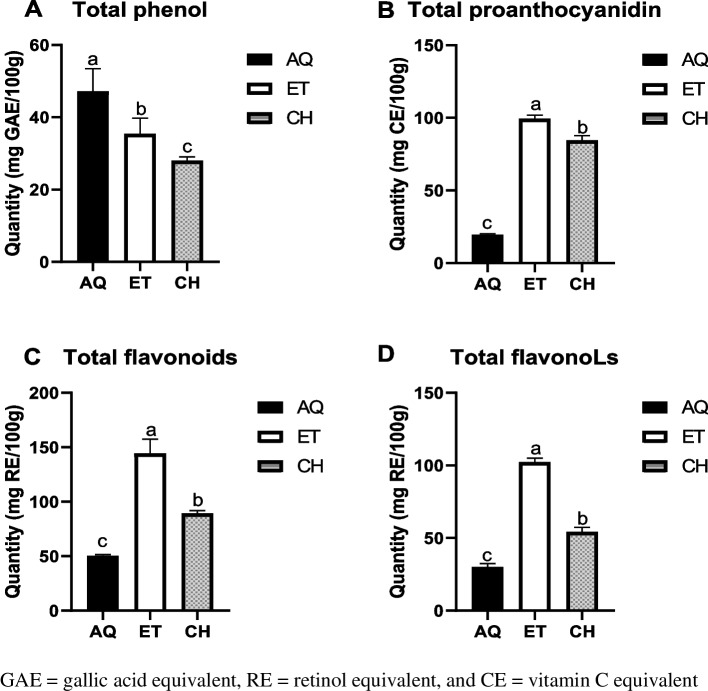
Quantitative phytochemical analysis. GAE, gallic acid equivalent; RE, retinol equivalent; and CE, vitamin C equivalent

The results for the qualitative and quantitative analysis of MWL are shown in Table 2 and Fig. 2, respectively. Phenolic compounds, flavonoids, tannins, triterpenoids, reducing sugars, and cardiac glycoside were present in any of the aqueous, ethanolic, and chloroform fractions of MWL extract. While saponins and terpenoids were only present in aqueous and ethanolic fractions, alkaloids were only present in chloroform fractions, and then steroids and anthraquinones were conspicuously absent in all three fractions of MWL extract. Interestingly, alkaloid was only present in the chloroform fractions, whereas on the quantitative analysis, the aqueous fraction (AQ) of MWL extract produced the highest amount of total phenol, followed by the ethanolic fraction (ET), and the ethanolic fraction gave the maximum quantity of total proanthocyanidin, flavonoids, and flavonols followed by chloroform fraction (CH). The MWL fractions vary significantly by ^a,b,c^*p* < 0.001, < 0.01, and 0.05.

**Table 2:** Qualitative phytochemical contents of MWL extracts

Phytochemical	Aqueous	Ethanol	Chloroform
Phenolic	+	+	+
Flavonoid	+	+	+
Saponins	+	+	-
Tannins	+	+	+
Steroids	-	-	-
Alkaloids	-	-	+
Anthraquinones	-	-	-
Terpenoids	+	+	-
Triterpenoids	+	+	+
Reducing sugars	+	+	+
Cardiac glycoside	+	+	+

+Present, -absent

**Fig. 3:**
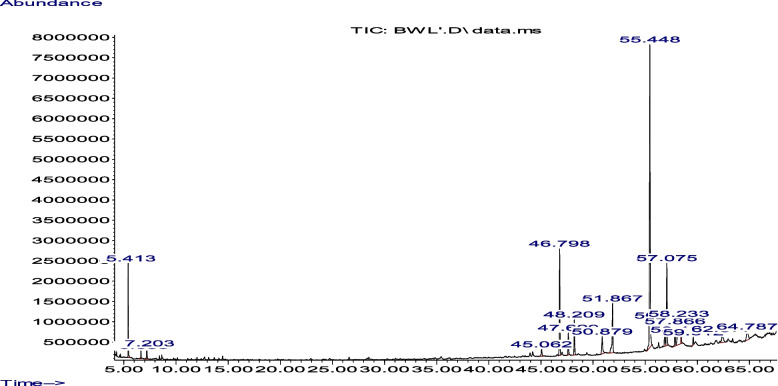
The GS-MS characterization of MWL. The peaks were displayed against the abundance and time axes of the GS-MS graphical representation with the highest peak at the time of approximately 55.5 and an abundance of 8,000,000

Figure 3 and Table 3 showed the GC-MS profile of the *Mondia whitei* leave extracts.

**Table 3:** GC-MS bioactive components of MWL ethanolic extract

Pk no.	RT	Area%	Library/ID	Ref. no.	CAS no.	Quality
1	5.413	4.54	Ethanone, 2-bromo-1-phenyl-	63952	000070-11-1	50
			Benzoyl bromide	51665	000618-32-6	40
			Benzeneacetic acid, alpha-oxo-, methyl ester	35628	015206-55-0	40
2	6.638	0.5	Heptane, 1,1-dimethoxy-	32548	010032-05-0	42
			Isobutanol, TMS derivative	22643	018269-50-6	17
			Dodecane, 1,1-dimethoxy-	93306	014620-52-1	12
3	7.203	0.94	Propanamide, N,N-dimethyl-	4170	000758-96-3	27
			Acetic acid, diethyl-	8255	000088-09-5	17
			4-Methyl-3-hexanol acetate	31275	084612-71-5	10
4	45.062	1.04	3-(4-Fluoroanilino)-1-(3-nitrophenyl)-1-propanone	147690	350039-84-8	74
			(S,E)-4-Hydroxy-3,5,5-trimethyl-4-(3-oxobut-1-en-1-yl)cyclohex-2-enone	85362	039763-33-2	64
			Phenol, 3-isopropoxy-5-methyl-	36241	1000221-46-6	58
5	46.798	10.84	Neophytadiene	138502	000504-96-1	94
			9-Octadecyne	111836	035365-59-4	62
			Bicyclo[3.1.1]heptane, 2,6,6-trimethyl-	17379	000473-55-2	60
6	47.629	2.3	Neophytadiene	138502	000504-96-1	89
			1-Methoxy-3-(2-hydroxyethyl)nonane	66682	070928-44-8	83
			9-Octadecyne	111836	035365-59-4	62
7	48.209	3.67	Neophytadiene	138502	000504-96-1	89
			1,4-Eicosadiene	138503	1000131-16-3	46
			trans-2-Dodecen-1-ol	51300	069064-37-5	43
8	50.879	2.81	n-Hexadecanoic acid	117419	000057-10-3	99
			n-Hexadecanoic acid	117418	000057-10-3	99
			n-Hexadecanoic acid	117417	000057-10-3	96
9	51.867	6.6	Hexadecanoic acid, ethyl ester	144309	000628-97-7	99
			Hexadecanoic acid, ethyl ester	144304	000628-97-7	98
			Hexadecanoic acid, ethyl ester	144308	000628-97-7	94
10	55.448	36.44	Phytol	155849	000150-86-7	99
			Phytol	155850	000150-86-7	87
			Phytol	155853	000150-86-7	87
11	56.883	2.82	Linoleic acid ethyl ester	167367	000544-35-4	99
			9,12-Octadecadienoic acid, ethyl ester	167389	007619-08-1	99
			Linoleic acid ethyl ester	167366	000544-35-4	98
12	57.075	8.92	Ethyl 9,12,15-octadecatrienoate	165627	1000336-77-4	99
			9,12,15-Octadecatrienoic acid, ethyl ester, (Z,Z,Z)-	165643	001191-41-9	99
			9,12,15-Octadecatrienoic acid, ethyl ester, (Z,Z,Z)-	165642	001191-41-9	99
13	57.866	2.18	Octadecanoic acid, ethyl ester	171414	000111-61-5	99
			Octadecanoic acid, ethyl ester	171409	000111-61-5	99
			Octadecanoic acid, ethyl ester	171412	000111-61-5	98
14	58.233	9.9	Pregna-5,16-dien-20-one, 3-hydroxy-, (3,beta)-	173537	001162-53-4	87
			Pyrene, 1,6-bis(1,1-dimethylethyl)	173585	055044-29-6	80
			5-Androsten-17alpha-ethynyl-3,beta,17,beta-diol	173540	1000126-90-5	55
15	58.432	0.98	2-Methyl-3-propenylcyclopropanecarboxylic acid, methyl ester	28669	118316-01-1	18
			Acetamide, 2-phenyl-N-(o-tolyl)-	92243	040748-53-6	15
			Furan-2-carboxamide, N-ethyl-N-(3-methylphenyl)-	92283	1000308-20-3	14
16	59.612	0.76	Cyclopropanecarboxylic acid, 3-ethenyl-2,2-dimethyl-, methyl ester,trans-	28682	041977-59-7	35
			Methyl 2-furoate	11322	000611-13-2	25
			Acetophenone, 4-nitro-, 6-pyrazolylcarbonylhydrazone	133261	1000260-38-0	25
17	62.377	2.17	Lanosterol	249538	000079-63-0	55
			9-Octadecenamide, (Z)-	141027	000301-02-0	51
			9-Octadecenamide, (Z)-	141028	000301-02-0	49
18	64.787	2.58	Sebacic acid, di(2-ethyl phenyl) ester	243068	1000355-12-6	60
			[1,3,5]Triazine, 2,4-di(imidazol-1-yl)-6-phenyl-	148663	1000303-87-5	55
			1,4-Naphthalenedicarboxylic acid-,di(4-methylphenyl) ester	236667	1000197-27-7	43

### Antioxidant effects and ferric reducing power of fractions of MWL extract

These are ascorbic acid (AA), antioxidant 2,2-diphenyl-1-picrylhydrazyl (DPPH) free radical scavenging activity (A), nitric oxide (NO) scavenging activity (B), ferric reducing power, FRP, (C), and ABTS radical scavenging activity (D), *p* < 0.001.

**Fig. 4:**
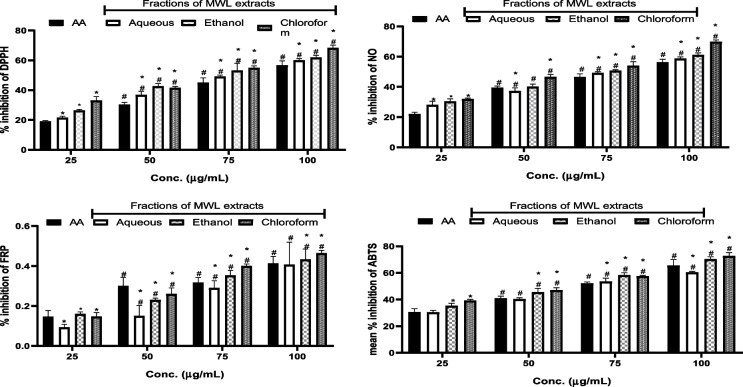
Binding pose of the potential inhibitors. Radical scavenging activities of MWL extracts

From Fig. 4 and Table 4, aqueous, ethanolic, and chloroform fractions of MWL and ascorbic acid gave the highest inhibition against ABTS, while ascorbic acid had the highest ferric reducing power, followed by chloroform, ethanolic, and finally by aqueous fractions of MWL.

**Table 4:** IC_50_ of MWL extracts in NO, DPPH, FRP, and ABTS

Samples	NO	DPPH	FRP	ABTS
Aqueous	37.09	36.61	0.15	40.14
Ethanol	40.09	42.46	0.23	45.53
Chloroform	46.54	41.24	0.26	47.00
Ascorbic acid	39.28	30.12	0.29	40.83

### Cytotoxicity effects of MWL extracts

**Fig. 5:**
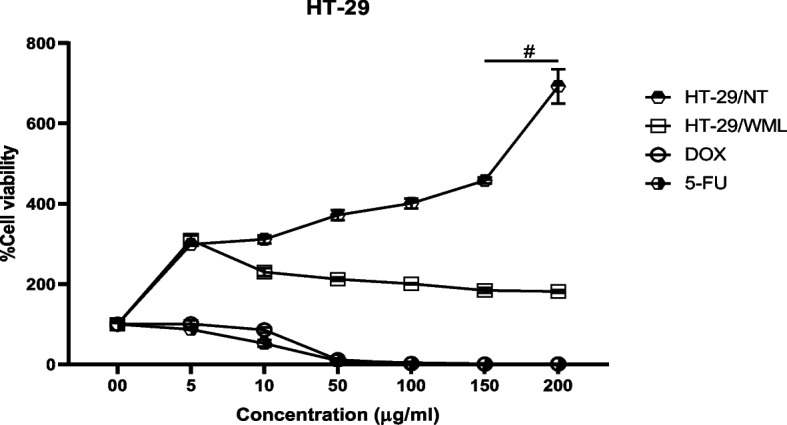
Binding interactions of the lead molecules at the binding pocket of CathB. Effects of ethanolic MWL extracts, doxorubicin, and 5-fluorouracil on HT-29 cell viability. HT-29 cells/no. of treatment (HT-29/NT), HT-29 cells/ethanolic extracts (HT-29/MWLMWL), doxorubicin (DOX), 5-fluorouracil (5-Fu)

**Fig. 6:**
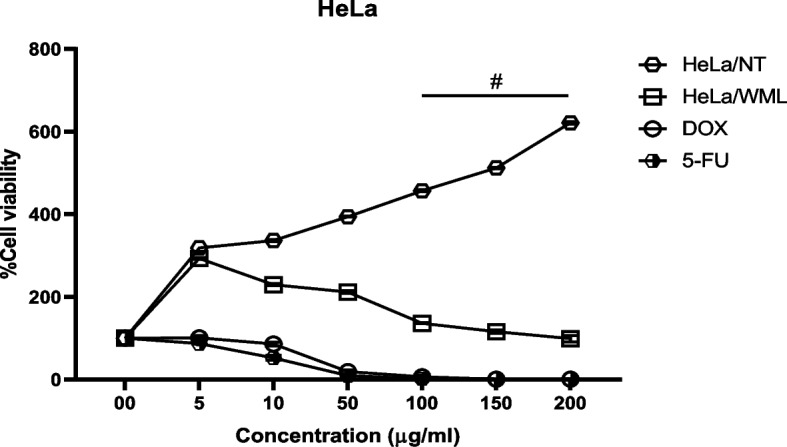
Radical scavenging activities of MWL extracts. Effects of ethanolic MWL extracts, doxorubicin, and 5-fluorouracil on HeLa cell viability. #*p* < 0.001 vs control; concentrations of doxorubicin and 5-fluorouracil are ×10^−1^ of their corresponding extracts

The cell viability of HT-29 and HeLa has substantially reduced at all DOX and 5-FU concentrations compared with the control, and the percentage of cell viability was oppositely correlated to DOX and 5-FU concentrations. The treatment of HT-29 and HeLa cells with MWL at concentrations of 150 and 200 μg/ml and 100, 150, and 200 μg/ml significantly induced cytotoxicity (^#^*p* < 0.001), while concentrations at 5, 10, and 50 μg/ml MWL have no significant effect on the cell viability Figs. 5 and 6.

### Target binding position and docking scores of potential inhibitors

**Fig. 7:**
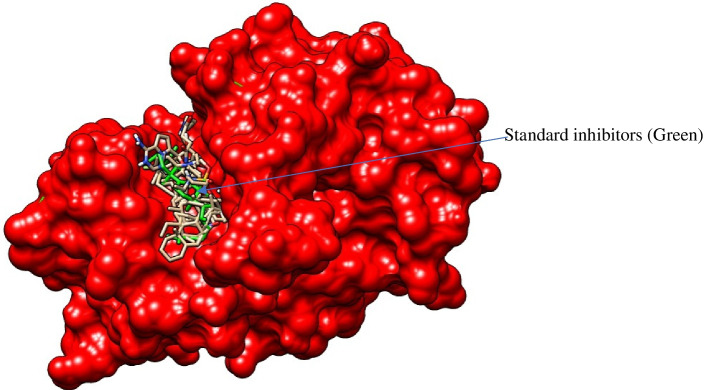
Effects of ethanolic MWL extracts, doxorubicin, and 5-fluorouracil on HT-29 cell viability. The 3D structure showing the competitive binding of the ligands within the pocket of Cathepsin B (visualized in UCSF Chimera)

Figure 7 shows the competitive binding between the potential and standard inhibitors. Table 5 shows the binding affinities of the ligand molecules.

**Table 5:** Docking scores (binding affinities) of the potential inhibitors

Given ID	PubChem CID	IUPAC nomenclature	Binding affinity (Kcal/Mol)
**CA-074**	6610318	Methyl(2*S*)-1-[(2*S*,3*S*)-3-methyl-2-[[(2*S*,3*S*)-3-(propylcarbamoyl)oxirane-2-carbonyl]amino]pentanoyl]pyrrolidine-2-carboxylate	−6.5
**MWL4**	580285	3-(4-Fluoroanilino)-1-(3-nitrophenyl)-1-propanone	−6.7
**MWL5**	10446	Neophytadiene	−5.0
**MWL6**	141998	9-Octadecyne	−4.4
**MWL8**	985	n-Hexadecanoic acid	−5.0
**MWL9**	121304016	Ethyl ester	−7.1
**MWL10**	5280435	Phytol	−5.2
**MWL14**	67032353	Pregna-5,16-dien-20-one	−8.2
**MWL15**	5368948	2-Methyl-3-propenylcyclopropanecarboxylic acid	−4.5
**MWL17**	246983	Lanosterol	8.3
**MWL18**	5192	Sebacic acid	4.8

### Target binding interactions of the lead molecules

**Fig. 8:**
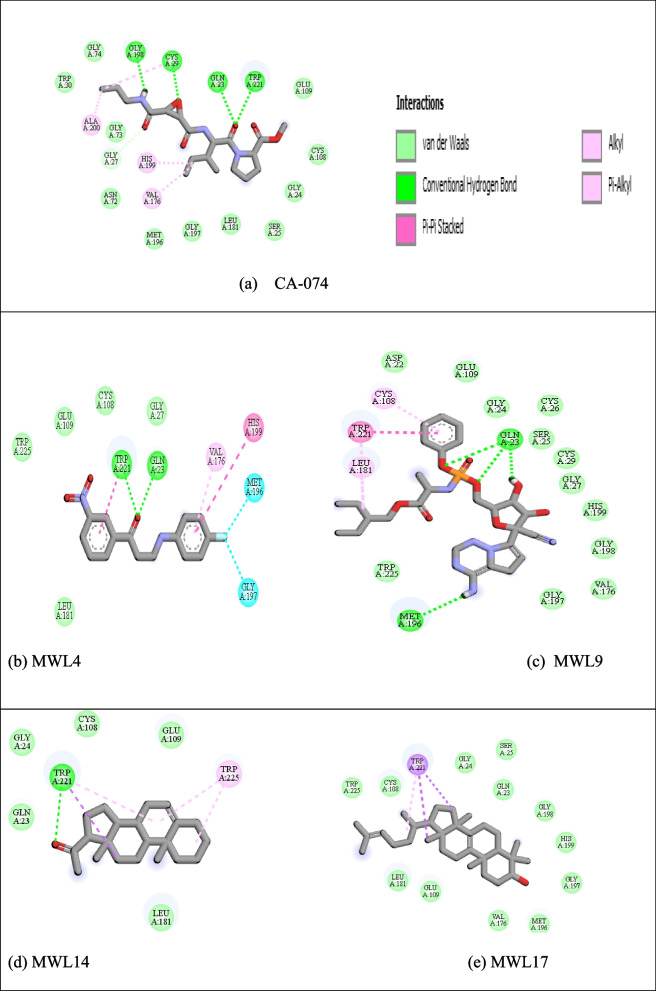
Effects of ethanolic MWL extracts, doxorubicin, and 5-fluorouracil on HeLa cell viability. The 2D structures showing the protein-ligand binding interactions and amino acids within the Cathepsin B pockets (**a** standard inhibitor; **b**–**e** potential inhibitors)

Figure 8 shows the binding interactions of the four MWL bioactives (MWL 4, 9, 14, and 17) with stronger affinities than the standard inhibitor. The bonds and the interacting amino acids within the pockets were also exposed.

## Discussion

In this study, the macro (essential)nutrients: sodium (Na), potassium (K), and magnesium (Mg) and micro (nonessential)nutrients: iron (Fe), copper (Cu), manganese (Mn), zinc (Zn), and nickel (Ni) were found in the leave extracts of *M. whitei* in varying degrees. Consumption of these macrominerals such as Na, K, and Mg and micronutrients such as Fe, Cu, Mn, Zn, and molybdenum (Mo) is very vital for specific physiological functions, while consumption of cadmium (Cd), nickel (Ni), chromium (Cr), lead (Pb), tin (Sn), and mercury (Hg) even in trace amounts results to toxic effects on the tissues of the body [37, 38].

The Amaechi and Egesi (2017) [48] study evaluates the nutritional values of the fruits of *M. whitei* (Hook. f.) Skeels consumed by some populations among the Izzi clan of Ebonyi state of southeastern part of Nigeria. The results found, among other components, the fruits contained a high amount of moisture (88.20%), total sugar (15.70%), reducing sugars (9.63%), nonreducing sugars (6.13%), the energy value of 40.80 KCal/100 g, very low riboflavin, thiamin (1.53 mg/100 g), and niacin (3.04 mg/100 g). The presence of antioxidants such as vitamins C (14.50 mg/100 g) and E (2.45 μg/g) and mineral elements with potassium and sodium being the most abundant. An insignificant amount of antinutritional phytochemicals which made them conclude that the fruit is not toxic for human consumption. In our study, sodium, potassium, and magnesium were found in the aqueous, ethanolic, and chloroform extracts as the macronutrients in the leaves of *M. whitei*. The lower levels of sodium and potassium found in this study suggest levels of mineral context may vary in the plant part (roots, stems, leaves, and fruits) compare to the report of Amaechi and Egesi (2017) [48] with the fruits of *M. whitei* having a higher proportion of sodium and potassium mineral elements. Further findings from this nutritional evaluation of the aqueous, ethanolic, and chloroform extracts of *M. whitei* leaves revealed the presence of varying amounts of micronutrients such as Fe, Cu, Mn, Zn, Cd, and Ni with lower concentrations of zinc and iron in the three leaf extracts of *M. whitei* studied than what was found in the wild edible fruit of *M. whitei* reported by Amaechi and Egesi (2017) [48]. While their study did not analyze copper, manganese, cadmium, and nickel contents of the wild edible fruits, our study did not analyze calcium and phosphorus.

Excess daily intake of sodium may lead to hypertension. However, the low levels of sodium found in the dry leaves of *M. whitei* (2.4–3.6 mg/100 g) did not occur at an amount that may lead to hypertension nor satisfy the daily sodium requirement of 1.2 to 1.5 g per day [48] assuming 300 g of the plant material, or equivalent of 70 g of the extract of *M. whitei*, is consumed daily. Consumption of an equivalent amount of the plant material (300 g) daily would also be too low (*K*: 1.9–4.0 mg/100 g; *Mg*: 0.2–0.5 mg/100 g; *Fe* (0.01–0.07 mg/100 g); *Cu*: 0.01–0.09; *Zn*: 0.01–0.02 mg/100 g; *Mn* (0.001–0.01 mg/100 g)) to provide sufficient quantity of the daily requirements of potassium (2.6–3.4 mg/day), magnesium (220–260 mg/day), Fe (17–20.5 mg/day), copper (2–3 mg/day), zinc (7–9.5 mg/day), and Mn (2–5 mg/day) [49, 50]. The work of Ngbolua and Mukeba (2020) [51] on the fresh leaves of *M. whitei* from northern Angola isolated larger amounts of K (1149.83 mg/100 g) and *Cu* 3.14 mg/100 g than in the dry leaves of *M. whitei* which were used in this present study, in addition to Ca (844.87 mg/100 g), P (175.89 mg/100 g), and *Se* 87.80 mg/100 g which were not analyzed in this study. The low level of these elements in our sample may be due to the loss of some of the nutrients from the leaves during drying and other processing activities or as a result of soil differences, as reported in the work of Janvier et al. [52], on the root bark of *M. whitei* obtained from five different localities in northern Rwanda which showed different macronutrients and micronutrient contents [52]. This suggests that it would be nutritiously auspicious to consume fresh leaves of *M. whitei* compared to the dry leaves, except in a situation where the components of these leaves could be concentrated and packaged for daily consumption according to the daily requirements of these micronutrients in the human body.

In this study, phenolic compounds, flavonoids, saponins, tannins, alkaloids, terpenoids, triterpenoids, reducing sugars, and cardiac glycoside were found present, while steroids and anthraquinones were absent in either of the three extracts (aqueous, ethanolic, and chloroform) of the dry leaves of *M. whitei*. Ngbolua et al. [53], in their mini-review, reported the presence of saponins, flavonoids, tannins, resins, and the absence of cyanogenic glycosides, anthraquinone, alkaloids, and cardiac glycosides in the leaves of *M. whitei*. This review complimented our study by confirming that the leaves of *M. whitei* contain saponins, flavonoids, and tannins, in addition to alkaloids, terpenoids, triterpenoids, reducing sugars, and cardiac glycoside not found in the study reported by Ngbolua et al. [53]. The difference might be due to soil differences or type of solvent used in extraction or the difference in ages of the plants used in both studies.

In other parts of the plant besides leaves, Inkoto et al. [54] reported that the roots of *Mondia whitei* contain phenolic compounds, coumarins, anthraquinones, anthocyanins, tannins, and iridoids using thin-layer chromatography, while Ndukui et al. [55] indicated the presence of saponins, phenols, alkaloids, and tannins in the ethanolic fresh root bark extracts of *M. whitei*. In the study conducted by Wacho et al. [56], reducing sugars, triterpenes, and steroids were found present in the aqueous and hexane extracts of *M. whitei*, confirming the presence of reducing sugars and triterpenes isolated from the leaves in the present study. Gbadamosi and Erinoso [57] reported in their study that the roots of *M. whitei* contained more saponins and tannins than in its leaves, while the leaves contained more flavonoids than the roots. However, in the wild edible fruits of *M. whitei*, Amaechi and Egesi [48] reported the fruits as containing tannins, phenol, phytate, sterol, carotenoid, oxalate, saponins, flavonoids, alkaloids and went further to quantify the amount of hydrogen cyanide (mg.kg^−1^) from the fruits of *M. whitei* on a fresh weight basis. All these reports of different parts of the plant containing different phytochemicals prove that phytochemical compounds vary according to the different parts of the plant, though similar in some instances. This variation in different parts of *M. whitei* could be due to certain environmental factors, seasonal variations, or differences in varieties of the species [58].

Food has been found to have a profound influence on health. This implies that the consumption of foods of high quality is necessary for body nourishment and protection against processes of inflammation and oxidative stress. Inflammatory and oxidative processes are healed or reduced by phytochemicals present in plant tissues. The use of the different parts of this plant to treat urinary infection, jaundice, headache and diarrhea, and other metabolic and infectious diseases in forms of the antioxidant property and ferric reducing power of MWL extract fractions. The free radical scavenging activity of MWL extract fractions was determined by nitric oxide, 2,2-diphenyl-1-picrylhydrazyl (DPPH), ferric-reducing power, and ABTS assays. The results showed three fractions of MWL extract (aqueous, ethanolic, and chloroform) exhibiting higher percentage inhibition than ascorbic acid against ABTS, respectively, and ascorbic acid displayed higher ferric reducing power than chloroform, ethanolic, and aqueous fractions of MWL extract. The results can be explained in favor of the ability of the different fractions of the MWL extract to effectively and efficiently scavenge for free radicals and mop them up, thereby inhibiting their deleterious effects in breeding inflammatory disorders such as cancer, arthritis, diabetes, hypertension, cardiovascular diseases, atherosclerosis, hyperlipidemia, obesity, aging, Alzheimer’s diseases, and other infectious diseases [59–62]. The high antioxidant propensity of the extract fractions indicates the positive correlation between the phenolic compounds such as polyphenol, flavonoids, flavonols, tannins, and phenolic terpenes in the leaves of *Mondia whitei* and their ability to scavenge for and inhibit the free radicals and their inflammatory and oxidative activities such as lipid peroxidation, protein, and DNA damage [56, 63–65]. Gbadamosi and Erinoso highlighted the superior antimicrobial and antioxidant activity of the aqueous extracts of both the roots and leaves of *M. whitei*, explaining the possible reason to be due to higher solubility of the active phytochemical constituents in the water [57]. This fact accentuates the expected effective role of the extracts of different plant parts in managing various metabolic and infectious ailments. This study assessed the antioxidant effects of phytochemical components of *M. whitei* and found that the aqueous, ethanolic, and chloroform fractions of the plant possess a high level of free radical scavenging activity against DPPH, NO, ABTS, and ferric-reducing power, which inflicts damage on macromolecules such as lipids, proteins, and DNA. It could be explained that the phenolic compounds in the leaves of *M. whitei* are responsible for this protection against macromolecular damage occasioned by the overriding influences of free radicals released by oxidative stress and metabolic processes in biological systems. This foregoing explanation is consistent with the fact that the aqueous leaf fraction contained the highest amount of total phenol and ethanolic fraction with the highest amount of flavonoids and flavonols, which have been reported by Ghosh et al. [66] to be invariably responsible for their antioxidant activity because of the hydroxyl group in the phenolic and flavonoid and flavonol constituents in the plant. The cytotoxic impact of DOX and 5-FU, well-known anticancer medicines, was confirmed by our investigation. The in vitro assay indicates MWL to have significant cytotoxic activity on HeLa and HT-29 cell lines due to the presence of mineral nutrients and phytochemical and bioactive components. The cell viability reduction of MWL agrees with the cytotoxic effect report of other medicinal researchers [45, 67–69] and opposite our previous study on the cytotoxic effect of *B. coriacea* Engl. (BC) on AsPC-1 cells [44]. However, it is important to identify the precise mechanism of action that triggers the cytotoxicity activity of MWL.

In the current study, in silico analysis was considered to gain insight into the molecular mechanism of anti-toxicity via antioxidant potentials of *M. whitei* leave extracts. Plant extracts have been explored following in silico studies [70, 71] to define the exact molecules underlying their therapeutic effects. We used a standard inhibitor to predict exact binding pockets. The choice of target protein was based on existing reports on overexpression of CathB in cancers [72] linked to oxidative damages. Cancers of the breast, cervix, bladder, stomach, colon, ovary, lung, prostate, and thyroid have all been associated with the overexpression of cathepsin B [73]. Ten of the eighteen bioactives identified in MWL showed potent inhibition of CathB revealing their molecular target with the binding energy of −4.4 to −8.3 Kcal/Mol. Interactions within the pockets revealed similar kinds of bonds. Although the molecular interactions revealed different amino acids in the pocket, we suggest that Trp221 and Gln23 could be the most critical target. For instance, all the potential inhibitors shared similar interactions compared to the standard inhibitors within the pocket, albeit the Trp221 and Gln23. Perhaps these amino acids play a critical role in the synthesis of nucleic acids translated to proteins like Cathepsin B in cancer cells to foster metastasis [74]. Again, malignant ovarian tumor cystic fluid had considerably greater cathepsin B and C levels [75]. Numerous investigations have shown that increased cathepsin B levels are associated with accelerated angiogenesis, invasion, and metastasis.

Furthermore, increased cathepsin B expression in the tumor epithelial cells of colorectal carcinomas was linked to a considerably reduced patient survival time [76]. Although stromal cells and normal epithelial cells nearby the tumors can express cathepsin B, the expression is often highest along the tumor’s advancing edge [77]. Interestingly, existing literature has demonstrated that amino acid restrictions play active roles in cancer interventions [78–80]. In the current study, we suggest that the inhibition of the amino acids in the binding pockets by the MWL bioactive can lead to reduced tumor vascularity, increased tumor cell mortality, decreased cell proliferation, and poor tumor invasion [81, 82]. Similarly, this has been reported in cathepsin B-deficient animals [83]. Therefore, we hypothesize that amino acid inhibition, especially in cancer cells, could be a critical molecular target of *Mondia whitei* leaves used to manage various diseases.

## Conclusion

The aqueous, ethanolic, and chloroform fractions of *Mondia whitei* leaf extract contain macronutrients such as sodium, potassium, and magnesium and micronutrients such as iron, copper, zinc, manganese, and nickel. These three extract fractions contained phenolic compounds, flavonoids, saponins, tannins, terpenoids, triterpenoids, reducing sugars, and cardiac glycoside, with aqueous and ethanolic containing higher contents of phenolic and flavonoid components and bioactive compounds, which were perhaps suggestive of being responsible for the observed antioxidant potentials of MWL. Findings from the in silico assay show that the bioactive components of *Mondia white* leaves extract inhibit Cathepsin B by blocking the amino acids in the pocket similar to the CA-074 methyl ester. The ability to inhibit CathB revealed by in silico assay and cytotoxic effects on cell lines suggest its potential effect on the cancerous cell. Therefore, we report that amino acid restriction is the potential molecular target of MWL extracts to elicit therapeutic effects.

## Data Availability

The datasets used and/or analyzed during the current study are available from the corresponding author on reasonable request.

## Abbreviations

ROS: Reactive oxygen species
OS: Oxidative stress
RNS: Reactive nitrogen species
NO: Nitric oxide
MWL: *Mondia whitei* leaves
UCHE: Uganda Council for Higher Education
DPPH: 2,2-Diphenyl-1-picrylhydrazyl
TCA: Trichloroacetic acid
CathB: Cathepsin B
